# The Meaning of Temporal Balance: Does Meaning in Life Mediate the Relationship Between a Balanced Time Perspective and Mental Health?

**DOI:** 10.5964/ejop.2415

**Published:** 2021-02-26

**Authors:** Jeffrey Dean Webster, Jonte Vowinckel, Xiaodong Ma

**Affiliations:** a Psychology Department, Langara College, Vancouver, British Columbia, Canada; b Department of Psychology, Clinical Psychology and Psychotherapy Section, University of Bonn, Bonn, Germany; c Department of Psychology, University of Houston – Clear Lake, Houston, TX, United States; University of Bari Aldo Moro, Bari, Italy

**Keywords:** time perspective, balanced time perspective, meaning in life, personality, eudaimonic well-being, hedonic well-being

## Abstract

The construct of a Balanced Time Perspective (BTP) predicts a variety of indices of mental health and well-being. We argue that one possible intermediate link between BTP and well-being may be an individual’s sense of presence of meaning in life. Participants ranging in age from 19 to 88 years (N = 192) completed two measures of time perspective (Zimbardo Time Perspective Inventory [ZTPI] and the modified Balanced Time Perspective Scale [mBTPS]), mental health, personality, and meaning in life. Correlational results showed that a BTP, mental health, and meaning in life were positively interrelated. Hierarchical regression models showed that a BTP (as measured with the mBTPS) explained additional variance in mental health beyond demographic, personality, and ZTPI scores. Mediation analyses showed that meaning served as a significant indirect link between BTP and well-being.

All experiences are filtered through the temporal lenses of past, present, and future: we remember earlier life events, live in the moment, and imagine events yet to come. These different time orientations may provide complementary pieces to both our overall well-being and our sense of meaning in life. Retrieving autobiographical memories may reinforce self-esteem, consolidate aspects of personal identity, and help us make sense of the choices and experiences we have lived to date (e.g., Westerhof, 2019); living in the moment may allow us to recognize what is meaningful to us, prioritize values, and express gratitude (e.g., Przepiorka & Sobol-Kwapinska, 2020; Vowinckel, Westerhof, Bohlmeijer, & Webster, 2017); and imagining the future may be a motivational source of hope, optimism, and the pursuit of purposeful goals (e.g., Phillips, 2018). As such, time is “…an integral part of virtually all psychological phenomena” (Carstensen, 2006, p. 1913), and “… one of the most powerful influences on virtually all aspects of human behavior” (Boniwell & Zimbardo, 2004, p. 167).

One robust area of time research concerns *time perspective* (TP), in which individuals demarcate the temporal flow of life into subjectively experienced past, present, and future. To date, the majority of TP studies reflect the current state of theory development, which is emergent, and therefore adopt an inductive approach to theory development. One general assumption from contemporary TP models asserts that although TP is influenced by various forces over the life course and can therefore manifest state-like qualities, adults generally adopt a particular temporal orientation which is relatively consistent over time (Boyd & Zimbardo, 2005). This trait-like quality allows for an investigation of individual differences in TP.

Additionally, recent research has focused on a *balanced time perspective* (BTP), which Zajenkowski, Stolarski, Witowska, Maciantowicz, and Lowicki (2016) state is a core feature of TP theory. A BTP is conceptualized as the ability to adaptively engage past, present, and future time perspectives in response to contextual forces. Consistent with this assumption, a BTP (as assessed using the Zimbardo Time Perspective Inventory: ZTPI; Zimbardo & Boyd, 1999), is associated with several positive psychosocial outcomes such as higher life satisfaction (e.g., Zhang & Howell, 2011), emotional intelligence (Stolarski, Bitner, & Zimbardo, 2011), psychological need satisfaction and gratitude (Zhang, Howell, & Stolarski, 2013), happiness (e.g., Boniwell, Osin, Linley, & Ivanchencko, 2010), and mindfulness (e.g., Muro, Feliu-Soler, Castellà, Deví, & Soler, 2017; Rönnlund et al., 2018; Stolarski, Vowinckel, Jankowski, & Zajenkowski, 2016).

Notwithstanding such findings, there are some potential psychometric and conceptual limitations in using the ZTPI for the purposes of assessing a BTP (e.g., McKay, Andretta, Magee, & Worrell, 2014; McKay et al., 2018; Stolarski, Wiberg, & Osin, 2015). In part as a response to some of these identified limitations, Webster (2011) originally developed the Balanced Time Perspective Scale (BTPS) consisting of past and future subscales. Subsequently, Webster and colleagues (Vowinckel et al., 2017) developed the modified BTPS (mBTPS) which added a present subscale. A BTP is described as a frequent and equal tendency to think about one’s past, present, and future in positive ways. Using the initial scale, a BTP was positively associated with self-esteem, happiness, attributional complexity, wisdom, and mental health (Webster, 2011; Webster, Bohlmeijer, & Westerhof, 2014; Webster & Ma, 2013), as well as higher delay of gratification and higher ability to imagine future scenarios (Gӧllner, Ballhausen, Kliegel, & Forstmeier, 2018). Recently, scores on a French version of the scale (Barsics, Rebetez, Rochat, D’Argembeau, & Van der Linden, 2017) positively correlated with emotional regulation and positive affect. Using the more recent mBTPS, Vowinckel et al. (2017) found that a BTP was positively correlated with measures of flow, mindfulness, and mental health and Webster (in press) found that a BTP was also positively associated with adaptive identity styles and flourishing. Together, these sets of findings provide compelling evidence for the relationship between various aspects of TP and positive mental health outcomes.

Unfortunately, many of these earlier studies did not include personality variables. This omission is important as “correlations between TP dimensions and SWB might be no more than a by-product of their covariance with traditional personality” (Stolarski & Matthews, 2016, p. 517). We address this earlier limitation in prior work by including a measure of the Big 5 personality traits in the current study.

An additional limitation in prior work investigating the association between TP and mental health concerns possible additional variables which might account for some of the observed covariance between these two constructs. We suggest that one plausible, and currently under-examined candidate, is the presence of meaning in life. Indeed, meaning has been theoretically predicted to be one possible outcome of psychologically healthy TP (Zimbardo & Boyd, 1999). Accordingly, we next describe research examining the relationship between TP and meaning in life followed by a discussion of the relationship between meaning in life and well-being.

## Time Perspective and Meaning in Life

We argue that meaning in life has a strong, perhaps necessary, temporal basis, and that all three temporal dimensions should be associated with meaning-making efforts. Consistent with this idea, Steger (2012), states that “meaning in life researchers presume they are studying a fundamental orientation of the person to the world, embracing all that is important and vital to someone’s past, present, and future” (p. 382). Given the multifaceted and complex nature of meaning (e.g., Heine, Proulx, & Bohs, 2006) it seems reasonable to suggest that the thoughts and emotions inherent in meaning making derive, at least in part, from different temporal frames (Vowinckel, Capaldi, & Passmore, 2016). Moreover, the meaning-TP dynamics can be modified by contextual factors such as serious health concerns (e.g., breast cancer; Martino & Freda, 2016).

With respect to a past orientation, meaning-making entails identification and understanding of earlier self-attributes, relationships, and developmental tasks. The task of identity formation and maintenance, for example, explicitly requires adolescents and emerging adults to remember earlier selves in order to see consistencies and differences between earlier and current self-concepts (Erikson, 1963). Moreover, there is an extensive literature on the benefits of reminiscence and life review (e.g., Pierce & Elliott, 2018) one of which is the consolidation of meaning (e.g., Webster, 2019; Westerhof, 2019). Autobiographical reflections can enable the emergence of thematic and coherent life narratives which have been related to greater meaning (e.g., Graci & Fivush, 2016; Webster et al., 2018).

Similarly, staying in the moment, or being present-focused can also contribute to greater meaning-making (e.g., Allan, Bott, & Suh, 2015). Rush and Grouzet (2012) for example, found that a positive focus on the present supported a mindful orientation. Mindfulness is a healthy, non-judging and open way of relating towards the present moment (e.g., Bishop et al., 2004), that supports knowledge of one’s true self (Carlson, 2013), which can be considered essential for a sense of a fulfilled and meaningful life (c.f. Schlegel, Hicks, King, & Arndt, 2011). Indeed, Allan et al. (2015) found that increased self-awareness mediated between mindfulness and meaning in life.

Finally, anticipating events yet to come and projecting oneself into an imagined future are processes related to well-being (e.g., Webster, 2011). Meaning-making is enhanced when specific future events (Waytz, Hershfield, & Tamir, 2015, study 3) are mentally present. Knowing where we are going in life, why these particular pursuits are worthwhile, and confidence regarding their achievement, provides a sense of direction and purpose (Emmons, 2003; Feldman & Snyder, 2005).

Theoretical discussion papers have suggested that a sense of meaning in life is related to either our past (e.g., Routledge, Sedikides, Wildschut, & Juhl, 2013; Sommer & Baumeister, 1998) or the future (e.g., Zaleski & Przepiorka, 2015). Despite this assumption, empirical studies investigating the relationship between meaning and all three TPs in the same study are sparse; consequently, the relationship between a BTP and meaning is still poorly documented and understood. In one study, meaning in life was associated with higher temporal extension, friendly attitude towards time and better temporal organization of behavior (Przepiorka, 2012). The author concludes that skills in time management and effective goal achievement may play an important role in meaning preservation in potentially difficult life periods such as young adulthood. Evidence from limited empirical studies using the ZTPI shows that meaning is positively associated with a positive past, and negatively correlated with a negative past, temporal orientation (e.g., Shterjovska & Achkovska-Leshkovska, 2014; Steger, Kashdan, Sullivan, & Lorentz, 2008).

The preceding empirical findings all lend support to the idea that a BTP contributes to a sense of meaning in life. More boldly, leading proponents of TP theory state that important social experiences “are assigned to temporal categories, or time frames, that help to give order, coherence, and *meaning* to those events” (Zimbardo & Boyd, 1999, p. 1271; italics added). It is this implied temporal precedence which we examine in the current study.

## Meaning in Life and Well-Being

Searching for and acquiring meaning is a fundamental motivation for humans (Frankl, 1984). Without meaning, life experiences are existentially compromised (e.g., Costin & Vignoles, 2019), and this state can contribute to higher levels of anxiety and depression. One definition of meaning in life is “…the sense made of, and significance felt regarding, the nature of one’s being and existence” (Steger, Frazier, Oishi, & Kaler, 2006, p. 81).

A large corpus of work is generally supportive of a positive meaning and well-being connection. For instance, the presence of meaning in life has been positively correlated with measures of love, joy, and life satisfaction (Steger et al., 2006), wisdom and growth narratives (Webster et al., 2018), and mental health (e.g., Perugini, de la Iglesia, Solano, & Keyes, 2017) and lower levels of health anxiety (Yek, Olendzki, Kekecs, Patterson, & Elkins, 2017). Meaning in life has also been shown to play a mediating role between variables such as gratitude and life satisfaction (e.g., Datu & Mateo, 2015), spirituality and mental health (Khumalo, Wissing, & Schutte, 2014), and knowledge of stroke and life satisfaction (Chow, 2017). These and other findings support Steger’s (2012) conjecture that meaning in life is “widely considered to be a critical ingredient in human well-being and flourishing” (p. 381).

Besides mindfulness, flow-experiences also likely contribute to a subjective sense of a fulfilled and meaningful life as individuals experiencing flow have a heightened sense of personal competence, satisfaction, and agency. Flow experiences can, therefore, lead to meaning making by ideally “transforming the entirety of life into a single flow activity, with unified goals that provide constant purpose” (Csikszentmihalyi, 2013, p. 213).

The present paper has two interrelated goals. The first goal is to add to the very limited empirical data currently available by investigating the relationship between a BTP and mental health and the possible role presence of meaning plays in this relationship. First, we examine bivariate associations among main study variables and predict that a BTP will be positively correlated with both overall mental health and the presence of meaning in life (H1). We also test mediation models in which we predict that the relationship between a BTP and mental health occurs in part indirectly through meaning (H2). It is important to acknowledge at the outset that cross-sectional data does not allow for causal statements. Our intention is to examine in a preliminary way one possible set of relationships among these variables. We return to this issue in the discussion section.

The second goal, given that the mBTPS is a relatively new measure, is to demonstrate its advantage over previously established measures, specifically the ZTPI, which we include in its entirety in this study for comparison purposes. We accomplish this goal by assessing the incremental validity of the mBTPS in a hierarchical multiple regression predicting that the mBTPS will account for unique variance in mental health (H3) after taking account of relevant control and ZTPI variables.

## Method

### Participants

We recruited 192 participants (142 females, 48 males, 1 person identified as “other,” and 1 person who did not report their gender) ranging in age from 19 to 88 years (*M* = 40.05, *SD* = 17.53, 1 person did not report their age). Participants were recruited in the United States from the general Houston, Texas area. The ethnic composition of the sample included 48.4% (White), 25.5% (Hispanic), 14.1% (African American), 6.8% (Asian), and 5.2% (other) participants in good health (*M* = 3.01, *SD* = 0.65 on a 4 point scale where 1 = *poor* and 4 = *excellent health*). The average education level was 14.74 years (*SD* = 4.57) and the project received ethics board approval.

### Measures

#### Presence of meaning

We assessed the presence of meaning in life by using the 5-item presence of meaning subscale (e.g., *I understand my life’s meaning*) of the Meaning in Life Questionnaire (MLQ; Steger et al., 2006). Responses were made on a 7-point scale where 1 = *absolutely untrue* and 7 = *absolutely true*. Cronbach’s alpha for this scale was .88.

#### Balanced Time Perspective (a)

The mBTPS is a 38-item scale containing two 14-item subscales, one reflecting a positive past orientation and one reflecting a positive future orientation, and one 10-item subscale reflecting a positive present orientation. Participants respond to each item on a 6-point Likert type scale where 1 = (*almost*) *never* and 6 *= always*. Sample items include: Past—*Tapping into my past is a source of comfort to me*; Present—*Being in the present helps me appreciate what I have*; Future—*I look forward to my future*. Subscale scores are averaged (in order to account for the lower number of items in the Present subscale) and then combined. Higher scores indicate greater BTP. Cronbach’s alpha for the past, present, future subscales, and total mBTPS are, respectively, .94, .91, .96, and .95.

#### Balanced Time Perspective (b)

The Zimbardo Time Perspective Inventory (ZTPI; Zimbardo & Boyd, 1999) is a 56-item scale which is constituted by the five subscales of past positive, past negative, present hedonistic, present fatalistic, and future. For better comparability of the two BTP measures, we operationalized BTP with the ZTPI as the deviation from balanced time perspective indicator (DBTP; Stolarski et al., 2011) multiplied with −1, so that the resulting score indicates the *proximity* to a BTP instead of the distance. Individuals rate on a 5-point Likert scale how strongly each statement applies to them (*very untrue of me* to *very true of me*). Examples of items are: *It gives me pleasure to think about my past* (past positive); *Painful past experiences keep being replayed in my mind* (past negative); *It is more important for me to enjoy life’s journey than to focus only on the destination* (present hedonistic); *My life path is controlled by forces I cannot influence* (present fatalistic); and *I believe that a person’s day should be planned ahead each morning* (future). Cronbach’s alpha in the present sample are: past negative (.84), present hedonistic (.74), future (.77), past positive (.78), and present fatalistic (.77).

#### Mental Health

We measured mental health with the Mental Health Continuum—Short Form (MHC-SF; Lamers, Westerhof, Bohlmeijer, ten Klooster, & Keyes, 2011). The MHC-SF assesses three domains of mental health, which are emotional, psychological, and social wellbeing. The rating instruction for the whole scale is: *During the last month, how often did you feel [...]*. Three items measure emotional wellbeing (e.g., *happy*), five items measure social wellbeing (e.g., *that you had something important to contribute to society*) and six items measure psychological wellbeing (e.g., *that you had experiences that challenged you to grow and become a better person*). Answer options range from 0 = *never* to 5 = *every day*. Cronbach’s alpha for emotional, psychological, and social well-being, respectively, was .84, .85, and .78. The total MHC-SF alpha was .90.

#### Personality

We used the Big Five Index (BFI: John & Srivastava, 1999) to assess personality. The BFI is a 44-item questionnaire in which participants respond to the stem, “I see myself as someone who…” on a 5-point Likert scale from 1 = *strongly disagree* to 5 = *strongly agree*. The BFI assesses levels of extraversion (“is talkative”), agreeableness (“is helpful and unselfish with others”), conscientiousness (“does a thorough job”), neuroticism (“can be tense”), and openness (“is original, comes up with new ideas”). Cronbach’s alphas were, respectively, .82, .70, .72, .80, and .76.

### Statistical Analyses

We first present basic descriptive statistics and zero-order, bivariate correlations among main study variables. We then present the results of a hierarchical multiple regression analysis testing for the incremental validity of a BTP as measured by the mBTPS for the dependent variable of mental health. Finally, we conducted a mediatiton analysis using Structural Equation Modeling (SEM) with AMOS (Version 26) software. We used SPSS (Version 23) to test correlational and regression predictions. Two persons were eliminated from the final sample due to excessive missing data. As missing data was very low (i.e., < 0.1%) it was considered missing at random and was replaced with mean imputation. Data was screened for normality. Tests for skewness and kurtosis were both within recommended ranges (i.e., < ± 1), and the Variance Inflation Factor (VIF) values were all < 2 indicating lack of multicollinearity. We took an additional step to ensure that our three main variables of interest (i.e., balanced time perspective, meaning in life, and mental health) showed sufficient discriminant validity by conducting the Fornell-Larcker criterion test. These results indicated that there was good discriminant validity among these variables in the current study.

## Results

As can be seen in Table 1, bivariate correlations are consistent with our predictions. Supporting H1, both BTP measures were positively correlated with mental health and the presence of meaning in life. The mBTPS and ZTPI correlated positively with each other. Interestingly, both BTP measures correlate higher with mental health than with each other, suggesting that somewhat different facets of TP are captured by each. We elaborate on this possibility in the discussion section. As personality, demographic, and both BTP measures were associated with mental health we conducted two hierarchical multiple regression analyses with mental health as the dependent variable to determine which of these classes of variables provided unique explanatory power.

**Table 1:** Means, Standard Deviations and Intercorrelations of All Variables

Variable	1	2	3	4	5	6	7	8	9	10	11	12	13	14	15	16	17	18	19	20
1. BTPS Pa	-																			
2. BTPS Pr	.47	-																		
3. BTPS F	.38	.49	-																	
4. ZTPI PN	−.16	−.25	−.20	-																
5. ZTPI PP	.59	.28	.18	−.30	-															
6. ZTPI PH	.17	.18	.09	.14	.13	-														
7. ZTPI PF	.01	−.09	−.31	.43	−.08	.34	-													
8. ZTPI F	.10	.25	.37	−.09	.21	−.18	−.36	-												
9. BFI E	.15	.14	.14^+^	−.19	.13	.15	−.11	.09	-											
10. BFI A	.20	.32	.12	−.27	.30	.00	−.19	.16	.08	-										
11. BFI C	.25	.34	.31	−.28	.32	−.15	−.35	.55	.19	.39	-									
12. BFI N	−.31	−.31	−.11	.52	−.36	.00	.27	−.05	−.29	−.39	−.41	-								
13. BFI O	.04	.25	.09	.03	.06	.27	−.12	.09	.26	.17	.20	−.08	-							
14. MHC E	.35	.44	.40	−.44	.35	.04	−.28	.15	.18	.25	.36	−.51	.03	-						
15. MHC P	.41	.50	.45	−.36	.38	.13	−.29	.32	.33	.22	.38	−.49	.20	.68	-					
16. MHC S	.35	.41	.19	−.28	.29	.08	−.11	.09	.15	.10	.19	−.38	.07	.50	.61	-				
17. MHC C	.43	.53	.39	−.42	.40	.10	−.26	.21	.25	.21	.35	−.53	.12	.83	.88	.85	-			
18. MLQ P	.39	.39	.40	−.26	.28	.08	−.23	.27	.36	.21	.37	−.30	.20	.45	.53	.43	.55	-		
19. mBTPS	.78	.81	.79	−.25^+^	.45	.18	−.16	.30	.18	.26	.38	−.30	.15	.50	.57	.40	.56	.50	-	
20. ZTPI	.38	.32	.33	−.76	.67	.02	−.57	.30	.23	.33	.39	−.50	.05	.49	.50	.31	.49	.33	.43	-
21. Age	.06	.04	−.34	−.11	.17	−.09	.00	−.17	.14^+^	.06	.12	−.33	.03	.15	.17	.28	.24	.13	−.11	−.10
*M*	4.12	4.44	4.92	2.93	3.58	3.29	2.35	3.74	3.40	3.95	3.86	2.91	3.62	3.95	3.92	2.85	3.57	5.35	4.49	−2.18
*SD*	0.97	0.86	0.96	0.81	0.65	0.53	0.70	0.58	0.76	0.56	0.58	0.79	0.60	0.85	0.90	1.09	0.81	1.20	2.22	0.77

*Note*. BTPS = Balanced Time Perspective Scale; Pa = past; Pr = present; Fu = future; ZTPI = Zimbardo Time Perspective Inventory; PN = past negative; PP = past positive; PH = present hedonistic; PF = present fatalistic; F = future; BFI = Big Five Index; E = extraversion; A = agreeableness; C = conscientiousness; N = neuroticism; O = openness; MHC = Mental Health Continuum short form; E = emotional; P = psychological; S = social; C = composite score; MLQ = Meaning in Life Questionnaire; P = presence of meaning; mBTPS = Balanced Time Perspective; ZTPI = Balanced Time Perspective.

Correlations ≥ .15 or .14^+^ are significant at *p* < .05; correlations ≥ .19 are significant at *p* < .01 and correlations ≥ .26 or .25^+^ are significant at *p* < .001.

We first entered the four demographic variables of gender, age, health, and education level as the initial block (see Table 2). Overall, model 1 was significant, *F*(4, 187) = 8.94, *p* < .001, and accounted for 16.0% of the variance in mental health.

**Table 2:** Hierachical Regression on Mental Health

Variable	Model 1		Model 2		Model 3		Model 4	
	β	*p*	β	*p*	β	*p*	β	*p*
Gender	0.009	.905	0.086	.191	0.087	.169	0.053	.351
Age	0.248	.001	0.109	.103	0.125	.054	0.197	.001
Health	0.223	.001	0.074	.259	0.057	.370	0.021	.713
Education	−0.192	.005	−0.155	.013	−0.125	.040	−0.089	.102
Extraversion			0.066	.318	0.043	.504	0.026	.655
Agreeableness			−0.048	.487	−0.081	.234	−0.095	.119
Conscientiousness			0.123	.079	0.082	.231	0.007	.914
Neuroticism			−0.422	.000	−0.327	.000	−0.283	.000
Openness			0.064	.313	0.076	.221	0.036	.517
ZTPI					0.266	.000	0.152	.021
mBTPS							0.420	.000
R	0.401		0.596		0.634		0.724	
*R*^2^	0.160		0.355		0.402		0.524	
Δ*R*^2^	0.160		0.194		0.047		0.122	
*F*_change_	8.94**		10.96**		14.24**		46.04**	

*Note.* mBTPS = modified Balanced Time Perspective Scale; ZTPI = Zimbardo Time Perspective Inventory.

**p < .001.

Model 2 added the five personality dimensions of extraversion, agreeableness, conscientiousness, neuroticism, and openness as a block. Model 2 accounted for an additional 19.4% of the variance in mental health and this change was significant, *F*_change_(5, 182) = 10.96, *p* < .001. Model 3 added the ZTPI score as a block. Model 3 accounted for an additional 4.7% of the variance in mental health and this change was significant, *F*_change_(1, 181) = 14.24, *p* < .001. In model 4 the mBTPS score was entered as a block. Model 4 accounted for an additional 12.2% of the variance in mental health and this change was significant, *F*_change_(1, 180) = 46.04, *p* < .001. In total, the four models accounted for 52.4% of the variance in mental health with age, neuroticism, ZTPI, and mBTPS as significant predictors.

Concerning a possible intermediate link between BTP and mental health via meaning in life, correlational analyses (Table 1) showed that both BTP measures, presence of meaning in life and mental health were positively interrelated. We used SEM to test whether meaning in life partially mediates the relationship between a BTP and overall mental health. Given the pattern of results of the hierarchical regression analyses, we also included age, neuroticism, and the ZTPI BTP score as control variables as these were the only variables to remain as significant predictors of mental health in the final regression model.

We used AMOS (v26) software with Maximum Likelihood estimation to assess parameter fit. We assessed overall model fit with the following standard statistics: χ^2^ goodness of fit test, the Comparative Fit Index (CFI), and the Root Mean Square Error of Approximation (RMSEA). Estimates of adequate fit vary, but conventionally a non-significant χ^2^, a CFI ≥ .95, and a RMSEA ≤ .06, are considered evidence of good model fit.

**Figure 1:**
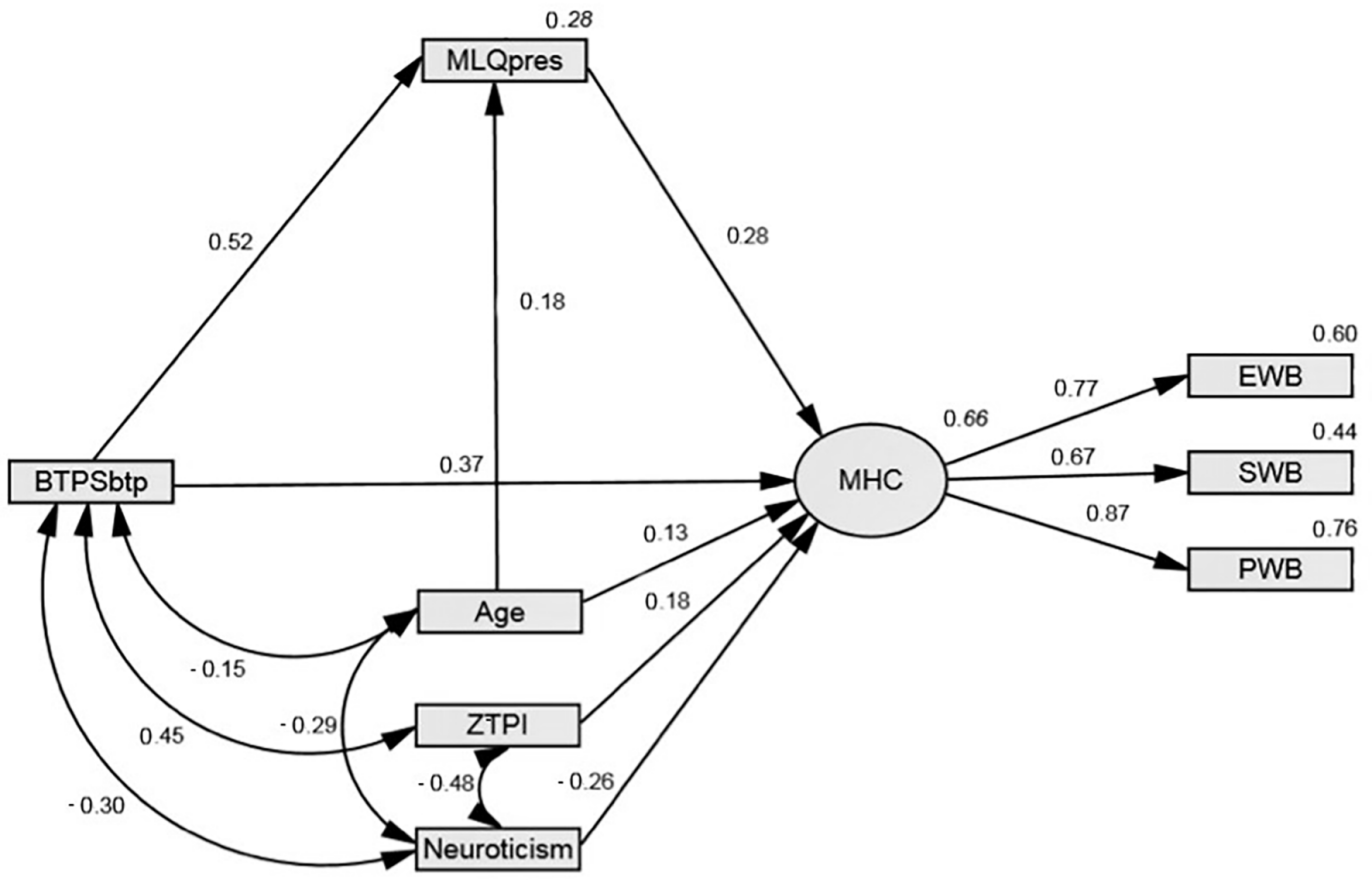
Meaning in life partially mediates the balanced time perspective to mental health relationship *Note.* BTPSbtp = modified Balanced Time Perspective Scale balanced time perspective score; MHC = mental health continuum; MLQpres = Meaning in Life Questionnaire presence of meaning subscale; ZTPI = Zimbardo Time Perspective Inventory balanced time perspective score; EWB = emotional well-being; SWB = social well-being; PWB = psychological well-being. Italicized numerals are *R*^2^ values.

Figure 1 shows the overall mediation model including standardized regression paths. Overall, the model explained 66% of the variance in mental health and fit the data well: χ^2^(13) = 21.60, *p* = .062; CFI = .985; RMSEA = .059.

We tested for mediation effects using a 95% confidence interval bias-corrected bootstrapping procedure with 5000 iterations. Table 3 lists the standardized direct effects.

**Table 3:** Standardized Direct Effects for Hypothesized Model

Parameter	Estimate	Bias corrected 95% CI		*p*
		LCI	UCI	
BTP to MLQ	0.521	0.388	0.623	.001
BTP to MHC	0.369	0.231	0.502	.001
MLQ to MHC	0.277	0.141	0.417	.001

*Note*. LCI = lower confidence interval; UCI = upper confidence interval; BTP = Balanced Time Perspective; MHC = Mental Health Continuum short form; MLQ = Meaning in Life Questionnaire.

As can be seen, all three direct paths were significant, satisfying the initial criterion for mediation analyses. We then used the *MyIndirectEffects.AmosEstimandVB* application (Gaskin, 2016) to test the indirect effect from a BTP to mental health via meaning. A significant indirect estimate is evidence for mediation, as is the case here. The indirect effect estimate was significant: estimate = 0.126, *p* < .001, LCI = 0.067, UCI = 0.196. As the standardized path from BTP to overall mental health remained significant after mediation, we can consider that meaning in life partially, rather than completely, mediates this relationship supporting H2.

Following best practices (Martens, 2005) we also tested an alternate model in which the direction of effect was reversed between a BTP and meaning, in which the meaning variable served as the predictor, and a BTP served as the mediator variable for the outcome variable of mental health. This alternate model did not fit the data well: χ^2^(13) = 45.98, *p* < .001; CFI = .941; RMSEA = .115. Although a χ^2^ difference test is inappropriate in the current situation (as the models are not nested; Bentler & Satorra, 2010) we did examine both the Akaike’s information criterion (AIC) and the Bayesian information criterion (BIC) indices whereby lower values are reflective of a better fitting model. These values also provided evidence for the hypothesized model having better fit than the alternate model for both the AIC (67.60 vs. 91.98) and BIC (142.52 vs. 166.91) indices, respectively.

## Discussion

In this study we investigated the relationship between a BTP and mental health as mediated by meaning in life. At the bivariate level, results confirm the positive association that a BTP has on well-being and engaging in life in a meaningful way. Supporting (H1), the mBTPS was positively correlated with all three facets of mental health (emotional, social, and psychological). Emotional well-being has been equated with hedonic facets of mental health and reflects a pleasurable engagement with life, a sense of satisfaction, and the attainment of at least normal levels of happiness. These results are consistent with prior work showing a positive link between TP and emotional (hedonic) well-being (e.g., Cunningham et al. 2015).

The findings also extend prior work by including aspects of well-being that go beyond just feeling satisfied or happy. Specifically, social and psychological well-being, in combination, have been equated with eudaimonic outcomes, ostensibly reflecting a deeper, more stable sense of well-being. As measured by the MHC-SF, social and psychological health derive from positive social engagement (e.g., contributing to others’ welfare) and the pursuit of growth experiences (e.g., challenging oneself to develop through important life events), respectively.

Also supporting H1, a BTP was positively correlated with meaning in life. Meaning-making is an ongoing process in which persons question their goals, priorities, and behaviors in relation to both existential and pragmatic challenges. What is my purpose in life? How do I fit into a larger cosmic order? Where have I come from and where do I need to go from here? The answers to these types of questions come, at least in part, from reflecting on prior experiences and imagining both anticipated and unexpected future life events. A BTP can serve as an important resource in this quest for purpose and meaning. In concert, these bivariate results suggest that finding a sense of meaning in life as well as attaining good levels of mental health can both be facilitated by a combination of a healthy balance of past, present and future perspectives.

In the hierarchical regression, a BTP, measured with the mBTPS, accounted for an additional 12.2% of the variance in mental health beyond the demographic, personality, and ZTPI variables indicating incremental validity for the mBTPS. In the final model only age, neuroticism, ZTPI, and mBTPS remained as significant predictors of mental health. Consistent with prior research, age was a positive predictor of mental health. Several studies (e.g., Thomas et al., 2016) have shown that despite increased levels of chronic illness, reductions in social network size, and possible cognitive decline, older adults show an “aging paradox” in that they maintain relatively high levels of well-being. Likewise, the finding that neuroticism is a negative predictor of well-being is consistent with a large and well-established body of earlier work (e.g., Sobol-Kwapinska, 2016).

The incremental validity results are noteworthy given that personality traits can act as confounding variables, often explaining sizeable amounts of the variance in independent and dependent variables. Moreover, the mBTPS accounted for unique variance even after accounting for an ostensibly similar conceptual measure (i.e., the ZTPI). These results are highly similar to Vowinckel et al. (2017) who used a Dutch sample. For instance, the correlation between the BTP and MHC-SF in Vowinckel et al. (2017) and the current study was 0.59 and 0.56, respectively constituting an important cross-national replication.

In mediation analyses, in line with Vowinckel et al. (2016), meaning in life’s indirect effect was significant in the relation between BTP and mental health. This effect was found for both BTP operationalizations and hence further promotes the hypothesis that BTP may positively affect mental health by facilitating an individual's sense of presence of meaning in life. The results suggest that meaning-making draws heavily upon temporal resources (e.g., positive motivational and affective qualities) which enable persons to engage in purposeful activities and make sense of their lives. Drawing upon consistently accessed and thematically organized personal knowledge enables persons to construct a comprehensible explanation for past, present, and anticipated experiences. This interpretation is supported by recent findings in which a BTP and a sense of coherence were positively correlated (Wiesmann, Ballas, & Hannich, 2017). In turn, having a clearly articulated sense of purpose and direction in life, contributes to enhanced levels of psychological, social, and emotional health.

### Conclusions

According to Boniwell and Zimbardo (2004), “discovering how to achieve a BTP should be a mandate for all of us. We believe it should be a central component in the agenda of positive psychology” (p. 165). The current project has provided modest, preliminary information in this regard; nevertheless, certain limitations need addressing.

First, given the correlational nature of our data we cannot make causal claims regarding directionality. Given the complexity of the constructs and possible conceptual overlaps, other models may produce similar results as well. It is possible, for instance, that higher levels of mental health cause increases in both meaning and the likelihood of developing a BTP, or that a BTP acts as a mediator between meaning in life and mental health. From a developmental perspective, however, this later possibility seems less likely as both meaning making and eudaimonic well-being are sophisticated psychosocial outcomes requiring high levels of cognition, self-reflection, and emotional maturity, attributes not typically seen until well into adolescence (e.g., Webster et al., 2018). Components of a BTP, in contrast, are seen much earlier in development. Basic reminiscence processes, for instance, are evident even in young children as young as 3 and 4 years old (e.g., Salmon & Reese, 2016); young children also demonstrate a preliminary understanding of the future (e.g., Atance & Mahy, 2016).

Nevertheless, with adult populations, as in the current study, it is probable that some type of reciprocal relationship may exist amongst these three variables and they may co-evolve in complex ways across the lifespan (e.g., Durayappah, 2011). Unfortunately, testing for such reciprocal relationships in SEM (i.e., a nonrecursive model) requires additional, stringent criteria such as equilibrium and stationarity (see Kline, 2011) which our current data set does not meet. Longitudinal studies are required to identify how these may mutually interact over time.

Second, although we included participants from diverse age-backgrounds, a limitation of the present study is its relatively small sample size. Although Kline (2011) notes the typical sample size for SEM studies is approximately 200 participants, a larger number of participants would have increased confidence in the findings. However, our sample size to model parameters ratio, while certainly not optimal, is adequate (Schreiber, Nora, Stage, Barlow, & King, 2006). Finally, as described earlier, our findings replicate previous work suggesting that the current results are not unduly jeopardized by sample size considerations.

Second, we note certain demographic limitations. First, the homogenous cultural background of our respondents reduces (intercultural) generalizability of the obtained results. We note, however, that our results are highly consistent with both Dutch (Webster et al., 2014) and French (Barsics et al., 2017) studies, both of which used larger samples (*N* = 512 and *N* = 622 respectively). Second, future studies should try to include a more balanced gender ratio as this study had a high percentage of female participants with higher levels of education.

Our results suggest many areas of future work. First, as meaning in life did not completely mediate the BTP/well-being link, it is very likely that other mediating variables are also involved in this association. For instance, a BTP might increase self-regulation/self-efficacy abilities (e.g., Fieulaine & Martinez, 2012; Lightsey et al., 2014) which may include heightened sense of personal agency, autonomy and competence. Inclusion of such variables in future studies will help fill the knowledge gap in this regard.

Second, future research can employ experimental approaches. For instance, TP might be experimentally manipulated by instructing participants to directly engage in thinking about their past, present, and future lives (e.g., in terms of remembered past accomplishments, valuable current projects, and anticipated future goals) to determine whether, compared to a control group, this manipulation increases the presence of meaning.

An additional area for future research relates to our secondary goal in the current project, namely the comparison between the ZTPI and mBTPS. Although the general pattern of findings between these two measures is similar, they represent different ways of conceptualizing and operationalizing a BTP. This is statistically demonstrated with the 0.43 correlation between them. Although this moderately strong correlation provides evidence of convergent validity, there is clearly a substantial amount of unaccounted for variance relative to each other. Additionally, several of the correlations between the two BTP measures and outcome variables were stronger than the correlation between the ZTPI and mBTPS. This also likely stems from the fact that the two measures are assessing somewhat different elements of time perspective. For instance, the ZTPI future score is often highly correlated with conscientiousness and seems to tap into concerns about obligations to perform future tasks at the expense of positive emotional experiences, as well as sometimes shows weak and/or non-significant correlations with aspects of subjective well-being like happiness (e.g., Simons, Peeters, Janssens, Lataster, & Jacobs, 2018). Future research comparing these two scales head to head could help identify other areas of divergence/convergence with the resulting information helping researchers when choosing a scale most appropriate for their research.

Despite the above limitations, our results contribute to the growing corpus of work on a BTP by replicating and extending earlier work on the relationship between a BTP and well-being and by investigating the relatively under-examined issue of how meaning plays an important role in this relationship. When persons frequently examine their positive past, present, and future in a balanced way, these combined temporal components provide a source of knowledge, solace, understanding, and direction to their lives. We argue this is a key, perhaps necessary, step on the road to discovering a meaningful life. When we know what makes life worth living and where we are heading, greater mental health is likely to ensue.

## Author Note

We dedicate this paper to our colleague, Xiadong Ma, who tragically died before completion of our project. The first and second authors contributed equally to the manuscript and authorship order was decided by consensus.
